# Impact of global health governance on country health systems: the case of HIV initiatives in Nigeria

**DOI:** 10.7189/jogh.05.010407

**Published:** 2015-06

**Authors:** Charles Chikodili Chima, Nuria Homedes

**Affiliations:** The University of Texas Health Science Center at Houston, School of Public Health, Houston, Texas, USA

## Abstract

**Background:**

Three global health initiatives (GHIs) – the US President’s Emergency Plan for AIDS Relief, the Global Fund to Fight AIDS, Tuberculosis and Malaria, and the World Bank Multi–Country HIV/AIDS Program – finance most HIV services in Nigeria. Critics assert that GHIs burden fragile health systems in resource–poor countries and that health system limitations in these countries constrain the achievement of the objectives of GHIs. This study analyzed interactions between HIV GHIs and the Nigerian Health System and explored how the impact of the GHIs could be optimized.

**Methods:**

A country case study was conducted using qualitative methods, including: semi–structured interviews, direct observation, and archival review. Semi–structured interviews were held with key informants selected to reach a broad range of stakeholders including policymakers, program managers, service providers, representatives of donor agencies and their implementing partners; the WHO country office in Nigeria; independent consultants; and civil society organizations involved in HIV work. The fieldwork was conducted between June and August 2013.

**Findings:**

HIV GHIs have had a mixed impact on the health system. They have enhanced availability of and access to HIV services, improved quality of services, and strengthened health information systems and the role of non–state actors in health care. On the negative end, HIV donor funding has increased dependency on foreign aid, widened disparities in access to HIV services, done little to address the sustainability of the services, crowded out non–HIV health services, and led to the development of a parallel supply management system. They have also not invested significantly in the production of new health workers and have not addressed maldistribution problems, but have rather contributed to internal brain drain by luring health workers from the public sector to non–governmental organizations and have increased workload for existing health workers. There is poor policy direction, strategic planning and coordination, and regulation of externally–financed HIV programs by the government and this poses a great limitation to the optimal use of HIV–specific foreign aid in Nigeria.

**Conclusions:**

A few reforms are necessary to improve the strengthening effect of GHIs and to minimize their negative and unintended consequences. This will require stronger leadership from the Nigerian government with regards to better coordination of externally–financed health programs. Also, donors need to play a greater role in addressing the negative consequences of foreign aid. The findings highlight important unintended consequences and system–wide impacts that get little attention in traditional program evaluation.

The immense suffering that has characterized the AIDS pandemic and other disease epidemics in low and middle–income countries led to the emergence of several global health initiatives (GHIs) in response to these deadly diseases. These GHIs have brought significant attention and massive resources to global public health [1]. For example, although donor funding generally constitutes less than 10% of total health care expenditures in Nigeria, the majority of AIDS spending – up to 85% in most years – has been donor funded [2,3]. Three Global Health Initiatives (GHIs) – the US President’s Emergency Plan for AIDS Relief (PEPFAR); the Global Fund to Fight AIDS, Tuberculosis and Malaria (Global Fund); and the World Bank Multi–Country HIV/AIDS Program have contributed most of these external funds [2,3]; more than four billion US dollars since 2001 (**Table 1**). In their effort to scale up services for diseases of interest such as HIV/AIDS, GHIs can either support countries to strengthen their weak public health systems in general (horizontal approach) or take a focal approach that prioritizes service delivery for the disease of interest, including the establishment of parallel health systems and processes if necessary (vertical approach). The latter approach dominates the GHI global health funding architecture [4]. Hence, despite their successes in scaling up critical services for targeted diseases, GHIs have been criticized especially for unintended consequences attributable to their vertical orientation and the resource constraints in the recipient countries. They have been noted to burden weak health systems in the recipient countries, by bypassing existing country systems and creating parallel and duplicative processes [1,5,6]. Secondly, the inherent weaknesses in the recipient country health systems, such as inadequate health care infrastructure, health workforce shortages, and poorly developed supply chain management systems, health information systems, and governance architecture limit the ability of GHIs to achieve their objectives [1,5,7–9]. Furthermore, while they might strengthen the capacity of health systems to respond to the HIV/AIDS epidemic, they might weaken their ability to respond to the needs of the entire population by diverting scarce resources to a particular disease area. Rather than fostering the unending age–long debate on horizontal vs vertical approaches to donor funding [4,10,11], empirical assessment of the impact of donor funding initiatives will be more helpful, to both donors and recipient countries, in identifying ways to optimize the impacts of such vital donor funds.

**Table 1:** HIV donor disbursements to Nigeria

GHI	Year of first grant to Nigeria	Disbursements (as at 31 March 2014)
World Bank MAP*	2001	US$ 221.48 million
Global Fund†	2003	US$ 433.91 million
PEPFAR‡	2004	>US$ 3.4 billion

*Source: World Bank Project and Operations – Nigeria, http://www.worldbank.org/projects/search?lang=en&searchTerm=&themecode_exact=88.

†Source: Global Fund Data Site, http://web–api.theglobalfund.org/DataAnalysts/Index.

‡Source: PEPFAR disbursement data are not publicly available. Figure is based on information from US Embassy Nigeria, http://nigeria.usembassy.gov/pepfar.html.

Empirical evidence of the country consequences of GHIs is limited and most of the single country studies on GHI–health system interactions have focused on individual GHIs, especially the Global Fund [12–15]. There has not been an in–depth assessment of the impact of GHIs on the Nigerian health system. Country situations differ widely in the sense that health systems are complex and their organization and performance are highly context–specific [1]; hence findings from other countries may not be entirely applicable to Nigeria. This study assessed the country–specific interactions and system–wide impact of the three aforementioned GHIs on the Nigerian health system, so as to provide information to policy makers and development partners on how to maximize the synergies between the GHIs and the country’s health system.

## METHODS

A country case study was conducted using qualitative methods, including: semi–structured interviews, direct observation, and archival review. Semi–structured interviews, lasting 45 to 90 minutes, were held with 36 key informants selected through purposive sampling, as well as by snowballing, to reach a broad range of stakeholders including policymakers, program managers, and service providers at different levels of the health system (federal, State, and local); representatives of donor agencies and their implementing partners (IPs); the WHO country office in Nigeria; independent consultants; and representatives of civil society organizations involved in HIV work (**Table 2**). Respondents provided written consent and all procedures were reviewed and approved by the research ethics committee of the authors’ institution and a locally relevant ethical board at the country of study. The respondents were assured confidentiality and anonymity; hence they were assigned a secret code that we used to identify the information and statements they provided. Several open–ended questions were asked of each respondent; however the interview guide was flexible to capture the unique knowledge of each interviewee. The interviews were conducted between June and August 2013, when permitted they were recorded, and otherwise handwritten notes were taken. The interviews were transcribed, coded using ATLAS.ti (v 7.1.7), and analyzed using a thematic analysis approach.

**Table 2:** Mapping of key informants

Stakeholder group	Number of interviewees
Government Representatives – Ministries and Agencies at the federal, state and local government levels:	
-Directors, policy makers, and other higher level managerial staff	4
-Project managers, program officers and other mid–level positions	8
Development partners: HIV/AIDS Global Health Initiatives and WHO Country Office	5
International NGOs/contractors	6
Local NGOs and contractors, and independent consultants	8
Advocacy groups	1
Public facility providers	3
Faith–based health care providers	1
Total	36

The findings from the interviews were checked against field notes from direct observation and extensive archival review. This triangulation minimized the potential for biases arising from recall failure or individual idiosyncrasy. The review included documents obtained through individual contacts and web–based searches. Included among these were strategic and operational plans, grant applications and project completion reports, and other relevant documents from organizations involved in HIV program funding and implementation in Nigeria; including the National Agency for the Control of AIDS (NACA), the Federal Ministry of Health (FMOH), National Primary health Care Development Agency (NPHCDA), the Global Fund, World Bank, PEPFAR, USAID, and The US Centers for Disease Control and Prevention (CDC). The approach also involved direct observation of meetings between implementing partners and government bodies, policy and planning meetings of government agencies, and visits to a couple of donor–supported Antiretroviral Therapy Clinics.

The case study approach is the best method for in–depth social science research [16] and was considered the most appropriate to extensively explore contextual influences on the interactions and impact of HIV GHIs on the Nigerian health system.

## RESULTS

Findings on the impact of donor–funded HIV programs on health system building blocks, as defined by the WHO, are presented below and summarized in **Table 3**.

**Table 3:** Analysis framework and summary of key findings

Health system building block	Themes	Major findings
**Leadership and governance**	System design, policy guidance and regulation, health sector accountability, civil society participation, dependency	• The country has not done a good job at coordinating donor funded programs for HIV • The coordinating infrastructure for HIV foreign aid is chaotic and not integrated with the health system • Because of the absence of strong policy direction, strategic planning, and regulation by the government, GHIs take a self–directed approach and do things as they deem fit • HIV GHIs have strengthened the role of non–state actors in health care • Donor funding has deepened a culture of dependency on foreign aid
**Health information systems**	Data availability, data demand and use	• HIV donor funding has strengthened information systems in the health sector • The culture of proper records keeping and data gathering has rubbed off positively on the system • HIV donor funding has improved the availability of good quality health information through population health surveys • Because of political constraints, improvements in availability of health data have not necessarily translated to increased utilization of data in program planning and implementation in the public sector
**Human Resources for Health**	Training; retention, distribution, and brain drain; workload, motivation and incentives	• GHIs have generally not invested significantly in the production of new health workers • PEPFAR is increasingly investing in pre–service training to improve the quality of health workers • The system is experiencing a training overload • The trainings are rarely evaluated for impact • Per diems have created disincentives for learning in the system: people go to trainings with the hope of ‘getting paid’ rather than to build their capacity • Activities of HIV GHIs have not positively affected the shortage of human resources for health in rural areas in tangible ways • A new trend in medical brain drain is emerging whereby health workers are lured away from the public sector to non–governmental or private sector organizations or projects funded by GHIs • HIV donor funded programs have increased workload for existing health workers by failing to invest in manpower recruitment • Though there are no salary differentials between health workers of the same cadre working on HIV programs and those working elsewhere, however those working on HIV programs typically have more opportunities for professional development and other benefits
**Financing**	Domestic allocations and sustainable financing	• Domestic allocations for HIV program delivery have generally been abysmal, as the government has practically handed over financing of HIV services to donors • Recently though, the President committed in July 2013 to scale up government’s financial commitment by launching the president’s comprehensive response plan (PCRP) for HIV/AIDS in Nigeria • The achievements made in HIV service delivery over the past decade is not sustainable as the current system cannot afford to continue deliver the services free of charge when donor funding ceases
**Service delivery**	Physical infrastructure, quality, equity and coverage, access and uptake, spillover effect	• HIV programs generally deliver services of higher quality than the rest of the system • An HIV donor funded initiative – The National Alliance for Health Systems Strengthening (NAHSS) – is working with the Federal Ministry of Health to develop a National Quality Improvement program (NigeriaQual) • Aid implementing agencies trade equity for efficiency when making service delivery decisions • Access to HIV services has increased but uptake has not been optimal • Best practices in patient care and follow–up in HIV program settings have impacted on other health services positively • HIV program scale–up crowded out delivery of non–HIV health service in the emergency phase of the AIDS response, however by strengthening health infrastructure HIV donor funds have also positively affected the delivery of other health services
**Supply management systems**	Procurement and distribution	• HIV GHIs have led to the development of a parallel procurement and supply management system • The elimination of fragmentation in the supply management system for HIV has reduced stock outs • The supply management system is not sustainable as it is run by a consortium of foreign technical organizations supported by donor grants

GHI – Global Health Initiative, PEPFAR – US President’s Emergency Plan for AIDS Relief

### Leadership and governance

Also known as stewardship, the leadership and governance function manages the other building blocks to achieve the objectives of the system. Key leadership and governance functions include policy guidance, regulation, system design, and accountability [17]; the impact of HIV donor funding is discussed along these lines.

**System design.** A crucial leadership function is to design the governance architecture in a way that ensures fit between strategy and structure and minimizes duplication and fragmentation [17]. As we will see, the stewardship of the Nigerian health system has failed to come up with an organizational design that achieves this purpose.

At the national level, there are several agencies responsible for coordinating development assistance and providing leadership for HIV programs. These are the National Planning Commission (NPC), National Agency for the Control of AIDS (NACA), the HIV/AIDS Division of the Ministry of Health (formerly the National AIDS/STI Control Program – NACSP), and the Global Fund Country Coordinating Mechanism (CCM) which oversees all Global Fund grants. To worsen the situation, these agencies and organizational units communicate poorly and their responsibilities overlap.

The NPC’s mandate is to manage and coordinate development aid and technical assistance from international development partners (Respondent 29 [R–29]). It approves initiatives that foreign entities want to conduct in Nigeria, including HIV programs. Unfortunately there is poor communication and coordination between the NPC and the line ministries; hence the interactions between donors working in the health sector and the NPC do not necessarily get communicated to the Ministry of Health and vice versa (R–10; R–29) (see Quote 1, **Online Supplementary Document(Online Supplementary Document)**).

Two major entities share the leadership of HIV programs: NACA and NASCP. NACA is tasked with coordinating the multi–sectoral response to HIV in Nigeria, which means bringing together the activities of the Ministry of Health, other ministries and government agencies, as well and the work of donor agencies and non–state actors to form one single ‘national response’ to HIV. NASCP on the other hand is tasked with overseeing the response of the health sector to the HIV epidemic in Nigeria, including granting approval to donor implementing partners (IPs) to carry out HIV programs in health facilities. Thus, there is overlap between the duties of NASCP and the NPC, and because of NACA’s overreaching nature (R–9; R–10; R–12) the roles of NASCP vs NACA are also unclear (R–12) (see Quote 2, **Online Supplementary Document(Online Supplementary Document)**).

The Global Fund requires recipient countries to establish CCMs to oversee Global Fund grants in their respective countries. However, the Principal Recipient (PR) of a Global Fund grant is the institution with whom the grant agreement is signed and remains the entity legally responsible for the execution of the contract [18]. The PR is responsible for grant implementation, monitoring and reporting and it is accountable to the CCM. By becoming a Global Fund grant recipient, thus making it accountable to the CCM, NACA is derailing from its coordination mandate and becoming more of an implementing agency.

**Figure 1:**
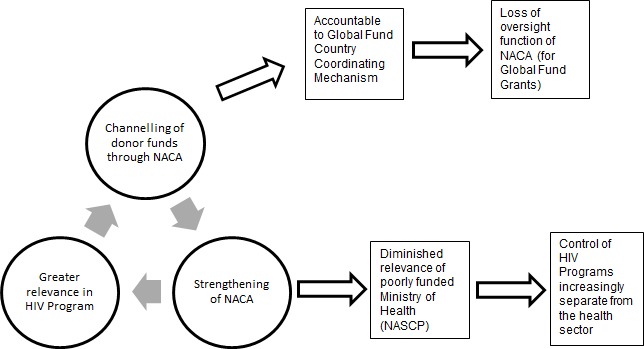
Impact of Channeling of Donor Funds through NACA on Governance of HIV Programs in Nigeria.

NACA and its equivalents at the State level – State Agencies for the Control of AIDS (SACAs) – have been greatly strengthened by donor funds, and they are now the most visible government players in HIV program delivery, while the Ministries of Health at the Federal and State levels increasingly lag behind (R–19; R–35). The idea of creating a body (NACA) independent of the ministry of health to oversee the national response to HIV was to overcome the bureaucracies in the ministry that militate against timely program implementation (R–2, R–19). However the creation of this parallel coordinating structure negatively impacts the sustainability of the programs (R–1) (see Quote 3, **Online Supplementary Document(Online Supplementary Document)**). In summary, the governance of HIV programs in Nigeria is chaotic (**Figure 1**).

For PEPFAR funds, PEPFAR agencies in Nigeria, namely the CDC country office, the USAID, and the US Department for Defense, are under the control of the country PEPFAR coordinator domiciled at the US Embassy in Nigeria. These institutions manage their own funds, activities, and processes (R–12; R–13; R–30).

**Policy guidance and regulation.** Collectively, the responsible government units have done a poor job at developing clear policies; formulating sector strategies; defining goals and directions; and identifying and managing the roles of donor agencies and their IPs, and other actors in the Nigerian health sector (R–9; R–12) (see Quotes 4 and 5, **Online Supplementary Document(Online Supplementary Document)**). The result of this government failure is that donors do things as they deem fit. For instance, the selection of facilities that will be equipped to deliver HIV services has mostly been based on where the donor agencies identify the right infrastructure for them to meet their project metrics in a timely manner (R–8; R–19; R–35).

The problem of poor donor coordination is even worse at the State level, as State governments have limited capacity for strategic planning (R–25) (see Quote 6, **Online Supplementary Document(Online Supplementary Document)**).

**Health sector accountability.** Generally speaking, donor funding for HIV programs has not had much impact on strengthening accountability in the public sector (R–5) (see Quote 7, **Online Supplementary Document(Online Supplementary Document)**). Though, by strengthening civil society, donor funding is contributing to building of mechanisms for checks and balances in the health system (R–25).

**Strengthening of civil society.** In general, HIV GHIs have strengthened the role of non–state actors in health care in Nigeria. Most of PEPFAR funding is channeled through non–state actors, although many of them are international non–governmental organizations (NGOs). The first round of HIV grant from the Global Fund was specifically designed to promote the effective participation of civil society organizations (CSOs) in the national response to HIV/AIDS. The World Bank has also promoted the participation of CSOs in the implementation of HIV programs [19]. CSOs help build accountability and keep the system honest through interventions in quality assurance and by independent confirmation of the data reported by HIV program implementing partners. For example, one of the CSOs – the Network of People Living with HIV/AIDS in Nigeria, NEPWHAN – uncovered gaps in access to HIV services at public facilities; including human resource issues like health worker absenteeism and impolite behavior (R–25).

Notwithstanding the positive contribution of CSOs, there have been legitimate concerns about insufficient accountability, legitimacy, and transparency within such organizations [20], and similar concerns exist in Nigeria (R–25) (see Quote 8, **Online Supplementary Document(Online Supplementary Document)**). The industry is poorly regulated.

**Dependency.** There is no doubt that donor funding for HIV has saved many lives in Nigeria but respondents believe that it has harmed the system by preventing the government from developing capacity and home–grown solutions to the HIV/AIDS epidemic (R–1; R–19; R–35). The role of GHIs is supposed to be to help fill funding gaps and provide limited technical assistance but some government units at different levels happily relinquish responsibility for the HIV epidemic to USAID and CDC (R1; R–12; R–19).

### Health information systems

A robust health information system (HIS) is one that ensures the production, analysis, dissemination and use of reliable and timely health information by decision–makers [17]. Here we document the impact of GHIs on data availability and data demand and usage by decision makers.

**Data availability.** Due to the absence of necessary data management systems, the electronic Nigerian National Response Information Management System (eNNRIMS) was created to pool and track data from health facilities to be able to monitor and evaluate HIV/AIDS services. There are ongoing efforts to integrate this system with the other data systems in the health sector to form a single national routine HIS that will become a go‐to source of health data, under the leadership of the government [21] (R–14). Hence, HIV funding could potentially strengthen the country’s HIS. At the moment however, integration has only being achieved within the HIV program; other programs – malaria, tuberculosis, etc. – have their own independent health information systems [21].

Besides the eNNRIMS District HIS, the culture of data gathering in compliance with monitoring and evaluation requirements for donor funded programs has rubbed off positively on the system. Most respondents attested to this fact (see Quote 9, **Online Supplementary Document(Online Supplementary Document)**). Non–routine health data sources, such as population health surveys, have also strengthened data collection efforts in Nigeria (R–14).

**Data demand and use.** Despite the increase in capacity to collect and interpret health metrics, improvements in availability of health data have not necessarily translated into increased utilization of data for policy formulation or health program planning in the public sector (R–19) (see Quote 10, **Online Supplementary Document(Online Supplementary Document)**).

### Human Resources for Health

Nigeria has several human resources for health (HRH) challenges including severe shortages and maldistribution. Shortage of qualified health workers in rural areas is particularly a problem [22, 23]. The critical shortage of health workers in Nigeria is worsened by a serious medical brain drain problem [22–25]. Low quality and quantity of human resources are known to militate against achievement of the objectives of GHIs [26], and there is concern that GHIs place significant burden on health care workforce [1,5]. We assessed the impact of donor–funded HIV programs on the availability and performance of health workers in Nigeria.

**Training of health workers.** Due to growing concerns that human resource shortages were limiting the impact of GHIs in developing countries, PEPFAR decided to finance the Medical Education Partnership Initiative (MEPI) – a five year health workforce development grant to 13 Medical Schools across Africa [26]. MEPI has a total worth, across all the recipient schools and countries, of US$ 130 million and is funded through the Office of the US Global AIDS Coordinator and the National Institutes of Health (NIH). The aim of the grant is to increase retention of doctors in the regions where they train and to address the persisting problem of maldistribution of health workers [26,R–5] by increasing the quality and quantity of medical graduates in the recipient schools and strengthening the research capacity of faculty by developing their ability to write and manage research grants. A consortium of six Nigerian medical schools applied for and is implementing MEPI. So far, MEPI has not increased the capacity for production of new health workers in Nigeria but it has impacted positively on the system in other ways, especially by strengthening research capacity [26,R–5] (see Quote 11, **Online Supplementary Document(Online Supplementary Document)**).

Another major way that HIV donor funding has impacted positively on the Nigerian health workforce has been through in–service training. Most of the trainings are focused on issues related to HIV treatment, although some deal with other issues such as financial management, supply chain management, and integrated service provision for primary health workers. Nearly all respondents pointed out this fact as a positive impact of HIV donor funding; however several concerns were also raised. Many of the respondents were of the opinion that the system is experiencing a training overload (see Quote 12, **Online Supplementary Document(Online Supplementary Document)**). A second issue is that the trainings are rarely evaluated for impact (R–12; R–28) (see Quote 13, **Online Supplementary Document(Online Supplementary Document)**). The finding of lack of evaluation of training programs was corroborated by a recently concluded assessment of in–service training of PEPFAR programs in Nigeria [27]. Third, the hotel–based nature of the trainings with their associated per diems has created disincentives for learning in the system. For many health workers, the trainings have become a way to top up their salary rather than avenues to improve their knowledge and skills. Thus people have developed ways to defraud the system (R–7) (see Quote 14, **Online Supplementary Document(Online Supplementary Document)**).

**Retention, distribution, and brain drain of human resources.** Maldistribution is one for the major challenges with health worker availability in Nigeria. A clinical program director noted that “Our problem is … retention and maldistribution. Many of the medical graduates leave and the ones that stay back reside in the urban areas, leaving rural people uncared for”(R–5). Activities of HIV GHIs have not positively affected the shortage of health workers in rural areas in tangible ways. This is unlike the situation in some other countries, like Zambia for example, where PEPFAR has funded a rural retention scheme that provides incentives to attract health workers to rural areas [28]. On the contrary, donor funded HIV programs in Nigeria have negatively impacted the maldistribution problem; in some instances State governments have redistributed health workers from facilities without donor‐funded projects to ones where projects are to be sited in order to meet the minimum requirement of the development partner (R–8) (see Quote 15, **Online Supplementary Document(Online Supplementary Document)**).

Also, the majority of HIV donor programs are situated in secondary and tertiary health care facilities, and these happen to be mostly in urban areas. Hence the programs might be further widening the divide between health care for urban and rural people.

Besides MEPI, there has not been much direct action by HIV GHIs to address the problem of poor health worker retention in Nigeria. On the contrary, most respondents agreed that with the advent of GHIs, a new trend in medical brain drain is emerging whereby health workers are lured away from the public sector to non–governmental or private sector organizations or projects funded by GHIs (see Quote 16, **Online Supplementary Document(Online Supplementary Document)**).

**Workload, motivation and incentives.** Generally, donor funded HIV programs do not cover the salaries of health workers. This is particularly the case for public sector facilities, where most of the respondents working for donor agencies or their IPs see it as the responsibility of governments to hire and pay health workers. In a few occasions however, IPs showed willingness to provide funding to private–for–profit and faith–based health facilities to augment their staff strength (R–16; R–27).

The few health workers in public facilities that receive donor support for HIV programs are left to bear the consequences of the increased workload consequent upon HIV treatment expansion without a corresponding increase in the number of human resources (R–5) (see Quote 17, **Online Supplementary Document(Online Supplementary Document)**).

There are no salary differentials between health workers of the same cadre working on HIV programs and those working elsewhere. However, those working on donor funded HIV programs typically have more opportunities for professional development through participation in capacity building workshops and, as mentioned earlier, monetary incentives are often given for such trainings in the form of per diems (R–9; R–13). The unequal incentive system has negatively impacted on collegiality and motivation in some instances, as people working on less funded programs felt less appreciated (R–9).

### Financing

**Domestic allocations.** The federal government piloted a national HIV treatment program in 2002 but never scaled it up, possibly due to the advent of PEPFAR and Global Fund shortly afterwards. As these GHIs became increasingly active in the country, the government handed over, almost totally, the financing of HIV services to these initiatives (R–1; R–5; R–17; R–19), with augmentation from two rounds of loans from the World Bank.

As a result since the year 2000, more than 85% of HIV expenditures have been donor funded. However it appears that the situation has begun to change, at least on paper. In August 2010, the Government of the United States (USG) signed a Partnership Framework on HIV/AIDS with the Government of Nigeria (GON) [29]. One of GON’s stipulated responsibilities in this agreement is to increase the proportion of GON financing for HIV/AIDS from 7% in 2008 to 50% by 2015. In line with this agreement, the President of Nigeria committed in July 2013 to scale up government’s financial commitment by launching the President’s Comprehensive Response Plan (PCRP) for HIV/AIDS. The PCRP has been praised as Nigeria’s own counterpart to PEPFAR (R–2, R–12, R–30). It aims to bridge gaps and establish a framework for achieving targets for HIV control by 2015 [30]. If fully implemented, it is projected that the PCRP will push domestic expenditure for HIV to 60% of total funding by 2015, but there is no evidence yet that the government is living up to this huge commitment.

**Sustainable financing.** The HIV delivery system has been described by many as a “*Cadillac System*” in the sense that donor funding has enabled the scale up of high quality services at minimal cost to the system and at almost zero cost to the end users in an environment where access to basic health services remains a big challenge. These short–term gains risk not be sustained if donor funding winds down. This is worrisome considering that globally, the growth rate of donor funding for health slowed dramatically in the recent past [31] (R–14). The sustainability issue was the elephant in the room throughout most interviews. Across the board, there was a consensus that the current aid–dependent model was not sustainable (R–1; R–19; R–24; R–25; R–28; R–30; R–35; R–36).

### Service Delivery

The impact of HIV donor funding on service delivery is presented under five important themes: physical infrastructure; quality; equity and coverage; access and uptake; and spillover effect on non–HIV health services.

**Physical infrastructure.** A significant portion of HIV funding has been invested in infrastructural support to health facilities, ministries of health, and other government agencies. These funds have been used to rehabilitate dilapidated buildings and build new ones, purchase vehicles, and develop Information and Communication Technology systems. Laboratories in donor supported sites have particularly benefited from HIV funding (R–5; R–7; R–11) (see Quote 18, **Online Supplementary Document(Online Supplementary Document)**).

**Quality.** Quality of health care is notably poor in Nigeria. The system is still dealing with fundamental issues of access to basic lifesaving interventions, and monitoring and enhancing service quality is not yet on the policy table. Nonetheless, HIV care and treatment stands out from the rest of the health sector due to the influence of the resources and monitoring systems made available through donor funding.

The facilities receiving support for HIV services from donor IPs are accountable to these organizations and receive supervisory visits from them (R–4; R–11); hence even in situations where things might not be working well in other sections of a hospital or clinic, providers have to maintain certain standards when they provide HIV services. This has resulted in better quality of HIV services and may have had a positive spillover effect on the system (see Quote 19, **Online Supplementary Document(Online Supplementary Document)**].

The Nigerian Alliance for Health Systems Strengthening (NAHSS), a PEPFAR–funded project that is being implemented through the CDC with the University of Maryland as a partner, is supporting the Federal Ministry of Health to develop a National Quality Improvement program (NigeriaQual). NAHSS aims to strengthen the capacity of local indigenous health organizations, States and health facilities to integrate quality improvement activities into organizational, financial and program planning activities, as well as into HIV care and treatment services at facilities [32] (R–20).

**Spillover effect on mon–HIV health services.** HIV programs have promoted the upholding of international best practices in HIV care and some of these have had system–wide impacts beyond the care of HIV patients. A good example is patient care coordination and follow up to improve adherence to treatment (see Quote 20, **Online Supplementary Document(Online Supplementary Document)**).

There is concern that the spillover effect of HIV could be weakening health systems in developing countries by diverting attention and scarce resources in the health sector, especially human resources for health, towards HIV programs [33]. In the case of Nigeria, some respondents felt that the HIV program was not big enough to lead to such effect (R–12; R–35) but a couple of them disagreed (see Quote 21, **Online Supplementary Document(Online Supplementary Document)**).

**Equity and coverage.** Aid implementing agencies were accused of caring less about ensuring equitable distribution of health services than about ensuring that they get good project numbers quickly, even at the expense of equity (R–8; R–19, R–35). This view was corroborated by the director of an international NGO (R–17) (see Quote 22, **Online Supplementary Document(Online Supplementary Document)**). It is ironical that the facilities that are doing better tend to be selected for more support, because such ‘viable facilities’ are more likely to quickly scale up services with minimal support (R–35) (see Quote 23, **Online Supplementary Document(Online Supplementary Document)**). This approach has resulted in gross inequities in the distribution of HIV services in Nigeria.

Another aspect of equity involves taking cognizance of and addressing socioeconomic barriers to accessing HIV services. Courtesy of donor funding, HIV treatment is provided mostly free at the point of service in most centers in Nigeria. Hence, affordability at point of service is not a major barrier to equitable access to HIV services.

**Access and uptake.** Although HIV/AIDS prevention, care and treatment services have dramatically increased, a persisting challenge is to attract the people who need these services to the facilities. As at December 2012, only 30% of the estimated 1.6 million Nigerians in need of antiretroviral therapy (ART) were receiving it [30]. In addition, a recently published UNAIDS report indicated that Nigeria has the largest number of children acquiring HIV infection from their mothers – nearly 60 000 cases in 2012 – and suggested that the country was not on track to meeting global targets by 2015 [34].

Respondents had different perspectives on the major reasons for poor uptake of the ‘free’ HIV services, including poor engagement of the private sector (R–28), poor awareness and insufficient demand–side interventions (R–21), and persisting stigma against people living with HIV (R–30).

### Supply management systems

The majority of HIV commodities, especially test kits and antiretroviral drugs, are purchased through PEPFAR or Global Fund support. The quantification of needs is done in unison for the entire country by all relevant stakeholders, including government agencies and development partners. After the quantification is done, PEPFAR and Global Fund then procure their share of supplies to fulfill the needs of all HIV programs in the country.

In Nigeria, PEPFAR procures HIV commodities through a project known as the supply chain management system, SCMS, whereas the Global Fund uses the Voluntary Pooled Procurement (VPP) system [35] (R–36). The procurement systems for HIV commodities are parallel to the procurement schemes for the rest of the health sector. The system for distributing HIV commodities is also dedicated to the HIV program and independent of the rest. Whereas most other commodities are moved from the federal level to regional/state level stores from where they are distributed to the facilities, the HIV program eliminates intermediary steps (R–31) (see Quote 24, **Online Supplementary Document(Online Supplementary Document)**). This challenge poses a limitation for integration with the supply chain system for other health commodities.

**The Supply Chain Management System (SCMS) Project.** The Partnership for Supply Chain Management, PFSCM, a consortium established by JSI Research & Training Institute, Inc. (JSI), and Management Sciences for Health (MSH) runs a PEPFAR–funded supply chain project for HIV commodities known as the supply chain management system (SCMS). Prior to 2012, various PEPFAR IPs were not only managing the implementation of HIV treatment services in facilities but also handling the distribution of HIV commodities. For better coordination and efficiency USAID/USG decided to unify supply chain management across its various partners. So SCMS now pools procurement for the various IPs and ensures distribution from manufacturer to the service delivery points (R–24; R–31).

After receiving approval from PEPFAR agencies, SCMS embarked on an exercise to bring the entire supply chain system for HIV commodities under its control, using a handful of strategically located regional hubs. Very importantly, the unification exercise was eventually transformed to become a national project, involving not only USG–affiliated organizations but also the Global Fund and the Government of Nigeria. So as it stands now, the distribution of HIV commodities for the entire country is handled entirely by SCMS. It also handles procurement for USG partners (PEPFAR financed) but the Global Fund still does its own procurement (R–31).

SCMS has significantly improved the availability of HIV commodities in Nigeria. Stock outs have been reduced appreciably (R–13, R–24), and it is likely that they have also harnessed economies of scale and improved on cost-effectiveness. However, SCMS has derailed from its original mission in Nigeria, which consisted of strengthening supply chain systems and building local capacity for logistics management through technical assistance (R–12; R–31). Instead the supply management system has become outsourced; rather than teaching locals how to do the job, SCMS has taken over the job (R–12; R–36). Most respondents familiar with the supply management system lambasted the current arrangement and stressed the need for a transition plan by which SCMS returns back to its technical assistance mission and transfers responsibility for operations to the public sector through the Federal Medical Store and the Federal Ministry of Health.

Despite its successes, the current procurement and supply chain management system is not sustainable (R–24; R–30; R–36) (see Quote 25, **Supplementary Online Document(Online Supplementary Document)**).

## DISCUSSION

### HIV/AIDS GHIs have had mixed effects on the Nigerian health system

HIV GHIs have had a mixed impact on the health system. They have enhanced availability of and access to HIV services, improved quality of services, and strengthened health information systems and the role of non–state actors in health care. On the negative end however, they have increased dependency on foreign aid; widened disparities in access to HIV services; done little to address the sustainability of the services; and led to the development of a parallel supply management system. They have also not invested significantly in the production of new health workers and have not addressed maldistribution problems, but have rather contributed to internal brain drain by luring health workers from the public sector to non–governmental organizations and have increased workload for existing health workers. There is poor policy direction, strategic planning and coordination, and regulation of externally–financed HIV programs by the government and this poses a great limitation to the optimal use of HIV–specific foreign aid in Nigeria. A couple of reforms are needed to improve the ability of HIV–specific foreign aid to strengthen the Nigerian health system. We look at these from the perspective of reforms needed on the part of the Nigerian government and the issues that donors need to address to improve the effectiveness of their investments.

### The Nigerian government should start leading

A major limitation to the optimal use of HIV donor funding in Nigeria is the fact that ‘the government is not leading’. Nigeria is an example of how poor coordination at the national level limits the ability of GHIs to strengthen health systems. As Vayrynen [36] once wrote, “…global governance cannot replace the need for good governance in national societies; in fact, in the absence of quality local governance, global and regional arrangements are bound to fail or will have only limited effectiveness”. The Nigerian government needs to do a better job at developing clear sector strategies and policies, identifying and managing the role of donor agencies and their IPs, and seeking avenues to increase domestic allocations for health. This is unlike the situation in some other African countries like Ghana, where the government sets clear policies with regards to development assistance for health, provides policy guidance, and regulates the activity of donors through a sector–wide approach that prevents parallel financing and delivery structures [12].

The first challenge is that control of the national HIV program resides outside of the health sector, causing duplication and wastage. NACA was established as a separate institution by the Presidency in a bid to achieve quick–wins in its response to the AIDS epidemic. Now that the emergency phase of the AIDS response is over, its future role should be addressed in the context of a general reform of the Federal Government that has a lot of parallel agencies and commissions that duplicate duties of ministries. The HIV/AIDS division of the Ministry of Health (NASCP) needs to be strengthened to play a central role in HIV program delivery in Nigeria; especially since most activities of the HIV program are based in the health sector.

Secondly, with regards to managing official development assistance (ODA), there is a need for better delineation of duties between the National Planning Commission (NPC), and line ministries and government agencies. The Ministry of Health should be given the responsibility for managing and coordinating ODA specifically intended for health programs. On the other hand, in conjunction with the Ministry of Finance, the NPC should be responsible for collating information on ODA across all government sectors and helping the executive arm of government to strategically incorporate ODA into national planning and budgeting. Clear communication processes and expectations should be set between NPC and ministries; one solution could be for each ministry to have liaisons at the NPC and vice versa, such that there is a constant channel for exchange of information regarding all ODA inflows and their utilization.

Thirdly, there is a dire need for strengthening health systems at local and regional levels. The country has a three–tiered system of government, namely Federal (National), State, and Local governments. For a country as big as Nigeria with 36 States, some with populations greater than many other African Countries, the importance of regional and local leadership cannot be overemphasized. Although HIV services have been significantly scaled up in the last decade as a result of which more than 500 000 Nigerians are now on antiretroviral therapy (ART) [30], the scale–up has not been done in an equitable manner and uptake has not been optimal. There are still 60 000 vertical transmissions of HIV in the country every year [34], and more than one million people living with HIV/AIDS eligible for ART are not yet on treatment [30]. There is need for a system–wide gap analysis at the state and local government levels to accurately map out areas and/or populations not being reached by services and devise strategies to address the gaps. Government leadership will be crucial in this regard and this will help donors to play a better role of filling the gaps in the system. State governments also need to do a better job of coordinating donor activities in their respective States for optimal outcomes.

The fourth needed reform is in the area of health information systems. HIV donor funding has improved the availability of good quality health data, however the reporting systems in the health sector are fragmented and would immensely benefit from integrating the various disease–specific platforms. Hopefully, ongoing initiatives in this direction will be sustained to ensure the establishment of a single robust health information system run by the Ministry of Health.

Finally, a plan for sustainability of HIV services in Nigeria needs to be articulated by the government, with donor support where possible. This will entail action in three major areas among others: increasing budgetary allocations, development of risk–pooling mechanisms for financial protection, and seeking market interventions to bring down the cost of HIV commodities to affordable levels. In April 2001, Nigeria and other countries in the African Union made a commitment to allocate at least 15% of their annual budgets to the health sector [37], however in the ten years following this pledge, Nigeria’s average government expenditure on health as a percentage of total government expenditure remained poor at 6.7% [38]. The recent launch of the Nigerian President’s Comprehensive Response Plan for HIV/AIDS, scheduled to run from 2013 to 2015, promises to increase the government’s contribution to HIV financing. The impact of this initiative on country ownership of the HIV program should be assessed in follow–up studies. An equally important sustainability issue is the need to address Nigeria’s lack of effective risk pooling mechanisms for health care financing. Donor agencies can provide financial and technical support to the government to strengthen and scale up the National Health Insurance Scheme as this will be crucial to the sustenance of the access to good quality services that have been promoted by GHIs. Finally, market–shaping interventions to increase access to essential health commodities, such as those championed by the Clinton Health Access Initiative and UNITAID, would go a long way in ensuring the ability of host governments to sustain HIV services in the event of decreased donor funding.

### Donors should invest more in systems strengthening and encourage country ownership

Donors have been criticized, and often rightly so, for caring more about achieving specific project–related metrics and less about the system–wide and long–term impacts of expenditures for health in recipient countries. Yet, it will take robust health systems to sustain the gains of billions of dollars of global health finances into the future. For example, the MEPI program which aims to strengthen health workforce in select countries in Africa only received 130 million dollars from PEPFAR contrasted to the billions it has spent on direct service delivery across the continent. As the prevailing dominant model of global health financing, GHIs can lead by example by prioritizing and emphasizing the strengthening of health systems in countries where they operate. In Nigeria in particular, the issues of access to health workers in rural areas remains a huge problem. GHIs should sponsor a rural retention scheme that provides tangible incentives to attract health workers to rural areas. There is also over–concentration of donor programs in secondary and tertiary facilities than primary health care facilities. The bias of secondary and tertiary health care towards urban areas mean that the rural–urban divide in access to good quality health services is further widened by donor intervention. Hence GHIs will do greater good by channelling increased resources to the strengthening of primary health care systems

Development jobs opened up by HIV donor agencies and their implementing partners may be helping to retain physicians and other health workers in the country, through the private sector. However, a good number of such jobs are non–clinical public health positions, so the impact on access to clinicians may be the same as if the providers had left the country. A more detailed study of this phenomenon would be necessary to characterize the magnitude and nature of the internal brain drain in other to proffer solutions. One approach could be by compensating the public sector for the internal brain drain by funding health worker recruitment and retention.

There is a need to conduct an impact evaluation of the health workforce in–service training programs. The few systems strengthening efforts of GHIs have focused heavily on such capacity building programs, yet there is a glaring absence of efforts to ascertain if and how these activities have improved the quality of health workers in Nigeria. Implementing partners should also transit from the current hotel–based in–service training approach to an institution based one whereby they collaborate with tertiary institutions and teaching hospitals to conduct on–the–job trainings without pulling the health workers away from their place of work. This will eliminate disincentives and resource wastages and refocus attention to a sustainable culture of continuing medical and nursing education.

GHIs should show more interest in the sustainability of the programs they finance by promoting country ownership. In Nigeria for example, PEPFAR should promote country ownership of the supply chain management system for the procurement and distribution of HIV commodities. The national unification project for HIV supply chain championed by SCMS has reduced fragmentation, increased efficiency, and decreased wastage. For these achievements to have lasting impacts beyond the duration of the contract for the SCMS project, JSI and its partners need to begin to lay greater emphasis on technical assistance to build the capacity of government staff at the Federal Medical Store in Lagos, and the various regional hubs and zonal stores. In 2012, a National Product Supply Chain Management Program was established under the department of food and drug services in the Federal Ministry of Health with the mandate to coordinate the logistics of various disease–specific programs in the health sector to ensure the minimization of stock outs and wastage of health commodities. This government unit, which is still in an infant stage, needs all the support possible from both the government and development partners so that it can grow and positively impact the system by building synergies across the various parallel supply chain systems for health commodities.

In conclusion, the impact of HIV GHIs on Nigeria’s health system has been mixed. This case study highlighted the importance of context in the debate about the system–wide effects of GHIs on country health systems, and offered practical solutions to some of the observed challenges.
