# Supplementary material for: Impact of global health governance on country health systems: the case of HIV initiatives in Nigeria
Source: J Glob Health. 2015 Apr 3;5(1):010407. doi: 10.7189/jogh.05.010407 (PMC4416331; doi:10.7189/jogh.05.010407)
Supplement: Online Supplementary Document [file jogh-05-010407-s001.pdf]

Online Supplementary Document

Chima and Homedes. Impact of global health governance on country health systems: the case of HIV initiatives in Nigeria

J Glob Health 2015;5:010407

Supporting Quotes

Quote number	Quote	Respondent number	Respondent background (type of organization)
1	"A lot of partners have direct access to our states and even to the Local Governments…. Once they get their papers [from NPC] they can go anywhere. … the national planning commission isn't really aligned with the health sector as they [sic] should be"	R-10	Federal Government (Ministry or Agency)
2	"The role of NASCP is to ensure that the policies, guidelines and protocols, and infrastructure are in place for effective service delivery and they also monitor to ensure that the services being provided are of acceptable quality. Unfortunately things are mixed up and the organizations are crossing boundaries into each other's roles and responsibilities. NACA has no business receiving grants and implementing. The roles need to be clearly defined."	R-12	Development partner/GHI
3	"A commission for civil service reform recommended the scrapping of NACA and many other agencies in the country due to the duplication and wastage of resources they cause"	R-1	Federal Government (Ministry or Agency)
4	"The country has not done a good job at coordinating donor funded programs… They should set the strategy and then request for help to fill in gaps where they might exist. I am yet to see a well-organized donor coordination meeting led by the FMOH or NACA."	R-12	Development partner/GHI
5	"What does not happen very often is NASCP or even NACA being able to direct and redirect efforts based on the analysis of the system to channel resources to where they are needed most… That is the main challenge… They are not in charge of it. Even NACA is not in charge of it. … when I talk coordination that is what I mean"	R-9	Federal Government (Ministry or Agency)
6	"There is lack of leadership and strong policy direction at the State level with regards to HIV program management.… The States have to play the leading role while the development partners key into the States' plans. Unfortunately as it stands at the moment, State governments do not have the capacity for such visionary leadership."	R-25	Civil Society/Advocacy Group

7	"Within the programs, accountability is high but I do not think that the health workers necessarily translate the lessons to other areas of the health sector that they might find themselves because of deeply entrenched systemic issues"	R-5	Academic/ Tertiary Hospital
8	"The civil society has had its own problems ... One thing I observed from my work is that many of the CSOs readily monitor and chastise other bodies implementing health programs but they do not want to be monitored nor let their processes be scrutinized by external parties... There is need for stronger regulation of CSOs and other organizations in the not-for-profit realm."	R-25	Civil Society/Advocacy Group
9	"HIV medicine has strengthened the entire system. You know that Nigeria has issues with paucity of health data, and this is because of poor culture of documentation. If you don't document, you cannot report. We [the IPs] have put protocols in place for proper data documentation"	R-11	Local NGO
10	"Training does not translate to usage. This is the case in Nigeria. Many people have been trained many times over but the system in which they operate does not encourage using data for decision making so their skills and knowledge are not put to any use…It is not that data is not being collected but the system is not there for them to be used... the State government that we are trying to strengthen does not do anything with the data."	R-19	Development partner/GHI (but formerly with State Government)
11	"On the implementation of MEPI, unfortunately we cannot do anything about [the] quantity of doctors that we are training as there are caps by the MDCN [Medical and Dental Council of Nigeria], based on the lecturer-to-student-ratio, facilities, etc. So you cannot go beyond that... Since we in Nigeria concluded that we cannot do anything about numbers, we decided to focus on quality by reviewing the curriculum for training medical students. By so doing we can improve [the] competence of our medical graduates… I think where MEPI can make a difference is the issue of quality of doctors…Another area of impact is the issue of capacity of medical practitioners to compete for research grants. For instance we did not even have eRA [Electronic Research Administration] registration. I am in the process of securing this for the University… and it was courtesy of MEPI. If you don't have eRA you cannot get NIH grants, so it is a big deal."	R-5	Academic/ Tertiary Hospital
12	"Too much attention is being paid to trainings; the system is obsessed with capacity building."	R-1	Federal Government (Ministry or Agency)
13	"A lot of training have taken place as noted, but the question is whether they [are] the right training given the situation. Some people have been trained several times on the same issues. We need to know if the lessons from	R-12	Development partner/GHI

	the trainings are retained by the trainees and how much the trainings have impacted the system, if at all. I do not believe that we have the right trainings to the scale that we should”		
14	“We have gone through phases where we paid so much per diem [and] we started cutting down. We had administrative person[nel] coming in for ART [antiretroviral therapy] training… Over time partners have realized that nepotism at the sites decided who went for trainings, so at a time partners started picking who goes for training.”	R-7	Local NGO
15	“Unfortunately in some instances what we have seen is redistribution of health workers by the government whereby they post health workers from facilities without donor-funded projects to ones where projects are to be sited, in order to meet the minimum requirement of the development partner. In such cases you have good outputs from project facilities at the expense of other communities with facilities that are not being supported by donor funds. So we cannot say that such approaches strengthen the health system.”	R-8	International NGO/Contractor
16	“I have seen many doctors and other health workers move from the public sector to international development programs. Many medical students now aspire for international development jobs. It has a lot to do with the remuneration and work environment. It is a fact that international development programs drain human resources from the public system. This is more worrisome when the doctors that move over to these programs assume non-clinical roles. Added to external migration, this could portend problem[s] for the system.”	R-19	Development partner/GHI (but formerly with State Government)
17	“They cannot just expect that since the machines are already here, they can just stretch us a little more to achieve their results. It doesn’t work that way. Some of us are already overstretched.”	R-5	Academic/ Tertiary Hospital
18	“Facilities that are supported to provide HIV care become strengthened to provide other services as well…. There is always a big difference pre and post the advent of HIV services at most facilities. We meet some of these facilities in very dilapidated states, and as you can imagine there is no way we can get operational without putting some basic things in place; for example basic amenities like water and electricity. And when we put these in place, it is definitely to the advantage of all patients.”	R-11	Local NGO
19	“Through this [HIV] program we have learnt the importance of quality assurance and the need for training and re-training of health workers.”	R-27	Faith-based Hospital
20	“…on the issue of home-based care, though we have had hospital visiting units for a long time they were barely active. But now the HIV-home based care people –	R-5	Academic/ Tertiary Hospital

	volunteer community health workers – do some serious work in maintaining contacts with patients and ensuring adherence to care. This improves retention rates… I think over time the learnings will diffuse to other health services. We are becoming more conscious of the need for follow up. …These elements are stressed in HIV programs and we, the health workers, are learning the importance of these issues.”		
21	“It diverted the attention of staff because they got stipends [and] they got all sorts of incentives for doing the HIV work to the detriment of the rest because we did not have an excess of human resource for health in the first place. So you find out that everyone wanted to be on the HIV program, even the non-health workers and I think this is one of the biggest problems.”	R-10	Federal Government (Ministry or Agency)
22	“the general approach in making decision for where to site treatment services trades equity for efficiency: we go for where we can reach the maximum number of patients”	R-17	International NGO/Contrac tor
23	“What has happened in most instances is that the IPs choose to site programs in facilities that have sufficient manpower to deliver the services they are bringing to the locality; where there is insufficient human resources they simply move on to viable facilities. There is documented evidence for this… IPs have been locating services only where things are working. This is a damning critique to the HSS [health system strengthening] work that has been done as part of PEPFAR; you cannot claim to be strengthening the system when you only choose to operate in facilities where things are already working well. It is a case of to him who has more, more will be given”	R-35	Federal Government (Ministry or Agency)
24	“…HIV is the only program whereby we plan for products to move directly from central to facilities. This is because ARVs [antiretroviral drugs] have short shelf lives”	R-31	International NGO/Contrac tor
25	“The current system is not sustainable considering that is expensive to run… I can tell you categorically that if SCMS were to pull out of the system right now, the supply chain system will crumble and there will be stock outs all over the country within two months”	R-36	Local NGO
